# *NRXN1-*related disorders, attempt to better define clinical assessment

**DOI:** 10.1515/med-2024-0979

**Published:** 2024-12-02

**Authors:** Piero Pavone, Xena Giada Pappalardo, Claudia Parano, Raffaele Falsaperla, Antonio Corsello, Enrico Parano, Agata Polizzi, Martino Ruggieri

**Affiliations:** Pediatrics, and Psychiatric Department of Child and Experimental Medicine, University of Catania, A.O.U. “Policlinico” “G. Rodolico”, Catania, Italy; National Council of Research, Institute for Research and Biomedical Innovation (IRIB), Unit of Catania, Catania, Italy; Department of Biomedical and Biotechnological Sciences (BIOMETEC), University of Catania, Catania, Italy; Department of General Surgery and Medical-Surgical Specialties of the University of Catania, Catania, Italy; Unit of Pediatrics, Neonatology and Neonatal Intensive Care, and Pediatric Emergency, AOU “Policlinico”, PO “San Marco”, Catania, Italy; Department of Clinical Sciences and Community Health, University of Milan, Milan, Italy; Department of Educational Sciences, University of Catania, Catania, Italy

**Keywords:** *NRXN1* gene, *NRXN1*-related disorders, seizures, Pitt-Hopkins syndrome, Pitt-Hopkins-like-syndrome 2

## Abstract

**Background:**

*NRXN1*-related disorders are uncommonly reported. The clinical features of the disorders are wide and heterogeneous mainly consisting of undistinctive facial dysmorphism, mild to severe intellectual and speech delay, epileptic seizures, and motor dysfunction. Defects in *NRXN1* gene have been identified in cases diagnosed as Pitt-Hopkins-like-syndrome 2 (PTHLS2; OMIM#614325).

**Methods:**

Literature review of *NRXN1*-related disorders was conducted and main clinical features of individuals affected by these disorders were analyzed. In addition, clinical features of individuals labelled with PTHSL2 diagnosis were reported. A comparison between international consensus diagnostic criteria for Pitt-Hopkins syndrome (PTHS) and twins presenting with *NRXN1*-related disorder and followed by this institution were also presented.

**Results:**

Our data confirmed that *NRXN1*-related disorders mainly manifest with undistinctive dysmorphic features and neurological involvement consisting of more or less severe developmental delay/intellectual disability, autistic spectrum disorder, and epilepsy. Relationship between PTHSL2 and *NRXN1* remains to be established.

**Conclusions:**

Our present analysis denoted a heterogeneous and unspecific clinical framework of the *NRXN1*-related disorders mainly affecting the nervous system for which the clinical diagnosis remains inconclusive without the support of genetic analysis. Further contributions are necessary to better clarify the clinical assessment of PTHSL2.

## 1 Introduction

Neurexin genes (*NRXN1, NRXN2, NRXN3*) code for cell-surface receptors that bind neuroligins to form Ca^2+^-dependent neurexin/neuroligin complex at synapses with a central role in synaptogenesis and vesicular neurotransmitter release [1–3]. Each gene isoform possesses two distinct promoters that begins the transcription from two separate locations: one known as α-neurexins, characterized by longer coding sequences leading to larger membrane-tethered components, and the other termed β-neurexins, featuring shorter sequences and consequently smaller membrane-tethered components [4–7]. Neurexin genes exhibit extensive alternative splicing at six canonical sites (referred to as SS1–SS6) [4]. The interplay of these exon-insertion splice sites generates thousands of spliced variants of neurexin proteins. This structural diversity dictates the binding specificities of neurexins to extracellular ligands, including neuroligins, leucine-rich repeat transmembrane proteins, cerebellins, and C1q-like proteins [3,8]. The alternative transcriptional program appears to endow with the ability to confer specific synaptic properties in different brain regions [9]. Therefore, gene-disrupting variants affect synapse function and neuronal connectivity leading to several phenotypic abnormalities, as described in human stem cell [10,11] and knock-out mouse model [12,13].

Mutations in the *NRXN1* gene (OMIM#600565) have been frequently detected in various neurodevelopmental disorders, particularly in individuals affected by severe developmental delay/intellectual disability (DD/ID), epileptic seizures, and autistic spectrum disorder (ASD) [14].

However, the extent to which genomic alterations of *NRXN1* contribute to the pathogenesis of these disorders remains uncertain [3,15].

In a wide systemic review of individuals with *NRXN1* deletions, Castronovo et al. [1] describe the presence of moderate to severe ID in 77–92%, ASD in 43–70%, attention deficit hyperactivity-disorder (ADHD) in 9–41%, anxiety in 6–7%, schizophrenia in 5%, epilepsy in 14–53%, and hypotonia in 38–47% of the cases. In addition, the authors highlight an elevated frequency of cases with receptive language delay and/or expressive language development disorder. Among 27 individuals presenting *NRXN1* deletions, Dabell et al. [16] report a clinical prevalence of DD particularly speech delay, abnormal behaviors, and mild dysmorphic features. Among this group, ASD was diagnosed in 10/23 (43%) and epilepsy in 4/25 (16%). Deletions of *NRXN1* gene have been also detected in individuals affected by Tourette syndrome [17]. Results obtained in individuals with deletion of *NRXN1* gene may show clinical signs sharing with the Pitt-Hopkins Syndrome (PTHS; OMIM#610954) and the affected patients have been classified under the term Pitt-Hopkins-like syndrome-2 (PTHSL2; OMIM#614325) [15,18–20]. The marked heterogeneity observed in genotypic and phenotypic manifestations among individuals with *NRXN1* deletion symptoms and PTHS highlights the need for further research to understand the similar and different mechanisms of pathophysiology [2,3,14].

PTHS is a genetic disorder mainly related to deletions or variants in the transcription factor 4 gene (*TCF4*; OMIM#602272) encoding a broadly expressed basic helix–loop–helix (bHLH) protein controlling the activity of many genes, most of which are involved in the nervous system development [21]. PTHS is characterized by syndromic facies, DD/ID, seizures, early-onset myopia, abnormal breathing patterns, anomaly in sleep–wake cycle, stereotyped behavior, and chronic constipations [20,22–25]. Diagnostic criteria of PTHS established by the first international consensus statement were reported by Zollino et al. [20] in which it is clear that the disturbed breathing regulation is one of the cardinal clinical element of PTHS cases. Recently, results of clinical correlation between *NRXN1*-related disorders and PTHSL2 have been questioned.

The present analysis aimed to analyze the main clinical features of the cases of *NRXN1*-related disorders reported by the literature. Clinical features presented by individuals affected by PTHSL2 were also discussed. Diagnostic criteria of PTHS dictated by Zollino et al. [20] were compared to clinical signs observed in twins affected by *NRXN1*-related disorders and described by one of our authors (R.F.) [26].

## 2 Methods

Literature review was conducted by collecting clinical trials, primary research, and review from online bibliographic databases (MEDLINE, Embase, PubMed, Cochrane Central, and Scopus) between January 2009 and January 2023. The key search derived from the medical subject heading terms were pertaining to children and “*NRXN1* deletions,” or “NRXN1-related disorders,” or “Pitt-Hopkins syndrome,” or “Pitt-Hopkins-like-syndrome-2.” Genetic studies, case-reports, and review articles were manually examined and included in the present reference list. After removing duplicate record, the main search results were included.

## 3 Results

We have collected the main clinical features of *NRXN1*-related disorders as described in 17 cases reported by the literature (Table 1) plus the twins by Sciacca et al. [26]. Analyzing these clinical results, we noted that undistinctive dysmorphic features was found in 16/17 (94%), DD/ID severe in 12/19 (63%), DD/ID mild 6/19 (31%) or moderate in 1/19 (5.2%), ASD 8/12 (66%), behavioral anomalies 14/17 (82%), epileptic seizures 8/19 (42%), and breathing regulation anomalies 2/17 (11%). MRI anomalies, heart, ocular, and skeletal anomalies were uncommonly reported. Mutations of *NRXN1* gene have been also reported in individuals with some clinical signs suggestive of PTHS and classified under the term PTHSL2 [15,18,19].

**Table 1:** Clinical features of 17 cases of *NRXN1*-related disorders reported by literature plus the twins by Sciacca et al. [26]

Clinical study	Gregor et al. [41]					Duong et al. [31]	Imitola et al. [42]		Viñas-Jornet et al. [43]		
No. cases	1	2	3	4	5	1	1	2	1	2	3
Genetic deletion size (kb)	103.5	324.3	72	1.5	144	451	130	107	417	627	516
Sex/age	F/4 y	F/4 y	M/8 y	M/15 y	M/11 y	M/33 y	M/7 y	F/7 y	F/21 y	M/20 y	M/11 y
Undistinctive dysmorphic features	+	+		**+**	+	+	nr	nr	+	+	+
DD/ID	Severe	Severe	Severe	Severe	Mild	Severe	Severe	Severe	Mild	Mild	Mild
ASD	nr	nr	nr	nr	nr	+	+			+	+
Behavioral anomalies	+		**+**	+		**+**	+	+	+	+	+
Seizures	+			+	+	+	+		**+**		
Breathing regulation anomalies	+							**+**			
Other features	nr	Hypotonia, MRI wide ventricles	nr	nr	Choanal and anal atresia	Cortical atrophy, cyst hippocampus	Ventricular septal defect	Strabismus ataxia	Dorsal kyphosis, long fingers	Dorsal kyphosis, finger rigidity	Undescended testicles, enlarged Virchow-Robin space

Clinical study	Holmquist [38]	Alfieri et al. [44]					Sciacca et al. [26]*		
No. cases	1	1	2	3	4	5	1	2	
Genetic deletion size (kb)	170	103.5	324.3	72	1.5	144	157	157	Ratio
Sex/age	M/4 y	F/4 y	F/4 y	M/8 y	M/15 y	F/11 y	M/12 m	M/12 m	12 M/7 F
Undistinctive dysmorphic features	+	+	+	+	+	+	+	+	16/17 (94%)
DD/ID	Severe	Severe	Severe	Moderate	Severe	Severe	Mild	Mild	Severe (12/19, 63%); mild (6/19, 31%); moderate (1/19, 5.2%)
ASD	+	+	+		**+**		nr	nr	8/12 (66%)
Behavioral anomalies	+	+		**+**	+	+	nr	nr	14/17 (82%)
Seizures				**+**			+		8/19 (42%)
Breathing regulation anomalies							nr	nr	2/17 (11%)
Other features	nr	nr	nr	nr	Gliosis alteration	nr	Triventricular hydrocephalus dilation	US IVH III grade	

Abbreviations: ASD, autistic spectrum disorder; DD/ID, developmental delay/intellectual disability; IVH, intraventricular hemorrhage; OFC, occipitofrontal circumference; US, ultrasound; nr, not reported; symbol “+” present; symbol “−” absent; m, month; y, year; *children diagnosed and followed by one of our authors (R.F.).

We have reported the main features of the patients labelled under the clinical diagnosis of PTHSL2 (Table 2) [15,18,19]. Mild facial dysmorphism was found in all, occipitofrontal circumference measurement was variable but within normal limits, DD/ID was severe in all individuals, 2/4 suffered from ASD, seizures was reported in 2/4, abnormal sleep–wake cycle in 2/4, constipation 3/4, breathing respiratory abnormalities and stereotypies in all the four individuals. In 3/4 patients, cardiac and pulmonary and others anomalies were reported.

**Table 2:** Clinical features of patients with PTHSL2 diagnosis

	Zweier et al. [15]	Harrison et al. [18]		Ruiz-Botero et al. [19]
No. cases	1	1	2	1
Age (years)	18	16	11	7
Sex	F	F	F	M
OFC	25th pc	79th pc	9th pc	60th pc
DD/ID	Severe	Severe	Severe	Severe
Seizures	−	+	+	−
ASD	+	nr	nr	+
Abnormal s–w cycle	nr	+	+	nr
Constipation	nr	+	+	+
Breathing anomalies	+	+	+	+
Facial dysmorphism	Mild	Mild	Mild	Mild
Stereotypes	+	+	+	+
Other features	nr	Pulmonary stenosis, early onset puberty	Mild scoliosis, early onset puberty	Left ventricular hypertrophy, pulmonary stenosis

Abbreviations: ASD, autistic spectrum disorder; s–w cycle, abnormal sleep–wake cycle; OFC, occipitofrontal circumference; DD/ID, developmental delay/intellectual disability; nr, not reported; symbol “+” affirmative; symbol “−” negative; pc, percentile.

In Table 3, clinical features between patients from literature with the diagnostic criteria of PTHS (first international consensus statement by Zollino et al.) [20] and twins affected by *NRXN1*-related disorders described by Sciacca et al. [26] and assisted in our institution are reported. No relevant similarities were found in the comparing data.

**Table 3:** Comparison of clinical features between patients from literature with the diagnostic criteria of PTHS (first international consensus statement by Zollino et al. [20]) and twins reported by Sciacca et al. [26]

Score of clinical diagnostic criteria for PTHS from Zollino et al. [19]	Sciacca et al. [26]*	
*Score ≥9. Molecular information indicated. Possible clinical diagnosis of PTHS*	Twin 1	Twin 2
*Score of 6–8. Presence of facial characteristics + additional criteria, either cardinal or supportive. This score warrants TCF4 molecular analysis. Insufficient clues for the presence of PTHS*		
*Score <6. No further studies specifically for PTHS indicated. Further studies for other etiologies indicated*		
1. Face		
a. Narrow forehead	−	−
b. Thin lateral eyebrows	−	−
c. Wide nasal bridge/ridge/tip	+	+
d. Flared nasal alae	−	−
e. Full cheeks/prominent midface	+	+
f. Wide mouth/full lips/cupid bow upper lip	+	+
g. Thickened/overfolded helices	+	+
2. Severe intellectual disability with absent or limited words speech (<5 words)	Moderate	
3. Breathing regulation anomalies (intermitted hyperventilation and/or apnea)	No	No
Supportive		
1. Myopia	No	No
2. Constipation	No	No
3. Hand (slender finger and/or abnormal palmar creases)	No	No
4. Unstable gait	+	+
Additional features		
Congenital symmetric brachycephaly	No	No
Curly hair	No	No
Primary teeth eruption delay	No	No

Abbreviations: − not investigated; + determined; no (absent sign/not determined); *children diagnosed and followed by one of our authors (R.F.).

## 4 Discussion

Among the studies regarding the clinical and genetic aspects of *NRXN1*-related disorders we examined 45 articles on this topic, 25 related to PTHS and 4 to PTHSL2. The clinical spectrum of *NRXN1*-related disorders is wide with neurological involvement as the main clinical features. PTHSL2 is a clinical entity linked to compound heterozygous mutations in the *NRXN1* gene, but the clinical boundaries with *NRXN1*-related disorders remain to be better defined.

Little is known about the human genetic regulation of *NRXN1* and mechanisms underpinning its high polymorphic expression as a result of alternative splicing [27]. Deletions or other genomic imbalances in *NRXN1* have been frequently associated with neurologic disturbances. However, the effects of *NRXN1* deletion on phenotypic characteristics are highly variable and mixed because they may involve minor isoforms, or those with unknown function, or fall within intronic regions not previously associated with a pathogenetic role [28]. Ching et al. [28] reported 12 subjects with deletion involving sequences of *NRXN1* ranging from 65 kb to 5 Mb. In this comprehensive study, the authors observed a diverse range in the age of diagnostic identification, spanning from 4 months to 16 years. They also delineated various inheritance patterns, noting maternal involvement in four cases, paternal in three, *de novo* mutations in four cases, and an unknown origin in one case. Furthermore, they identified dysmorphic features in eight subjects, while five were diagnosed with ASD, six exhibited cognitive DDs, and five displayed motor impairments. Behavioral features were noted in five individuals. Additionally, cardiac involvement was observed in three subjects, while none reported respiratory complications.

Schaaf et al. [29] found intragenic deletions of *NRXN1* in 24 patients, 17 cases involving exons and in the remaining 7 affecting introns. Almost all the individuals with exonic deletions showed DD/ID (93%), infantile hypotonia in 59%, and ASD in 56% of the cases, whereas congenital malformations and dysmorphic features were equally reported [29]. Mutations of exonic 1–5 deletions compared to 6–24 exonic deletions have been associated with low penetrance and have been also found in healthy controls [1,30,31].

The imperfect segregation of copy number variants (CNVs) may be linked to incomplete penetrance and variable expressivity as well as multifactorial interactions and modifying factors [32,33]. It may be assumed that the deletion in the first exons has probably a regulatory effect on gene transcription and influences the activation of alternative promoters mapped along the gene playing a compensatory role in healthy subjects [1–3,14,16]. The role of CNVs deletion in healthy parents remains to be established, although the inheritance pattern of incomplete penetrance of CNVs is usual in neurodevelopmental disorders and may not exclude the pathogenic link between the microdeletion and the disruption of gene regulatory elements leading to *NRXN1* expression in the neural circuits [29,34].

In a group of 25 patients with exonic deletions of *NRXN1*, Béna et al. [35] reported on a recurrent phenotype, features consisting of moderate to severe ID in 91% of the cases followed by severe language delay in 81%, ASD in 65%, seizures in 43%, and hypotonia in 38%.

Dabell et al. [16] describing the clinical features of 27 individuals with exonic *NRXN1* deletions stated that the exon deletion frequency is significantly higher (0.11%) than what has been found in control (0.02%; *P* = 6.08 × 10^−7^) suggesting a relevant role in the abnormal clinical development. *NRXN1* deletion is thought responsible for the clinical features presented by the affected individuals including craniofacial dysmorphisms, mild DD, speech delay, and other congenital anomalies. *NRXN1*-deletion has been associated with a wide spectrum of neuropsychiatric disorders [36], such as ASD and schizophrenia [37]; dysmorphic features and ID [38]; DD, short stature, and congenital diaphragmatic hernia [39]; psychosis and enlargement of the temporal horns of the lateral ventricle [40], speech and language delay as a consistent clinical manifestation [41–44]. Given the phenotypic heterogeneity, extensive studies are required to investigate the functional characterization of sequence and structural variants in *NRXN1*, aiming to elucidate the biological mechanisms, age-dependent and tissue-specific differential expression, genetic interactions, and neural circuits underlying both normal and pathological conditions [14].

## 5 Conclusions

To date, individuals with *NRXN1-*related disorders show to have a heterogeneous spectrum of clinical manifestation; therefore, a consistent clinical diagnosis is not easy to obtain and it remains linked to the result of the genetic analysis.

As shown by the results of the literature, the clinical features of individuals affected by *NRXN1*-related disorders show to have some but not all the classical signs of PTHS and PTHSL2 disorder. In fact, more recent observations have reconsidered the clinical correlation between *NRXN1*-mutations and PHTSL2. Therefore, a more specific ORPHANET entry addressed to the PHTSL2 (ORPHA 600663) seems justified.

In summary, examining the cases of *NRXN1*-related disorders reported by the literature it appears evident that the main clinical features consist of facial dysmorphisms and cerebral involvement with DD/ID as a prominent sign. Facial dysmorphisms lack distinctive features and thus do not contribute significantly to a clear diagnosis. However, cognitive impairment consistently emerges as a prominent and variable feature, ranging in severity across cases. Additionally, common observations include seizures, deficits in social communication and interaction, and behavioral anomalies. Breathing regulation anomalies, although infrequently reported, should not be overlooked. By gathering comprehensive family and medical histories and conducting thorough physical examinations focusing on facial dysmorphisms, DDs, and behavioral disturbances – especially indicative of ASD – genetic analysis emerges as the primary avenue for achieving an accurate and expedited diagnosis. Expanding clinical analysis to encompass multiple organ systems, including the heart, thorax, internal organs, and brain, is crucial as these areas may be affected.

Regrettably, there is no cure for this disorder. However, treatment should be administered, particularly addressing brain-associated anomalies such as epileptic seizures. Psychotherapeutic and pharmacological interventions should be tailored to individual clinical presentations, supplemented by educational and support services to optimize patient outcomes.

## 6 Future directions

Recent relevant studies have addressed the role of *NRXN1* mutation in pathogenesis and in treatment of some *NRXN1*-related disorders [45–47]. Data reported by Mu et al. suggest the involvement of the long noncoding RNA metastasis associated lung adenocarcinoma transcript 1 (*MALAT1*) in the pathogenesis of learning and memory disturbances in ADHD disorder [45]. They in fact analyzed a regulatory mechanism between the *MALAT1* and *NRXN1* through the interaction of two microRNAs, miR-141-3p and 200a-3p, establishing a so-called “*MALAT1*-miR-141-3p/200a-3p-*NRXN1*” axis, which impairs the synthesis of several synapse-related proteins in ADHD and could be used as diagnostic biomarkers. Another intriguing experimental study was conducted by Fernando et al. with separate populations of glutamate and GABA neurons pointing phenotypic distinction across glutamatergic and GABAergic neurons and identified phenotypic divergence across glutamatergic and GABAergic neurons [46]. According to their results, authors refer a design of novel therapeutic agents for *NRNX1* deletions on loss and gain-of-function stratified mechanism in affected individuals [46]. Ultimately, Maury et al. identified somatic copy-number variants (sCNVs) from 12,834 patients with schizophrenia and 11,648 as controls [47]. They also found sCNVs together with recurrent somatic deletions of exons 1–5 of the *NRXN1* gene in five schizophrenic cases leading to a potential role of sCNVs as a risk factor for schizophrenia [47]. All these findings provide important clues on the role of *NRXN1* and novel therapies in several diseases, encouraging further studies to investigate the regulatory patterns and tasks performed by *NRXN1* in brain development, thus helping to distinguish the phenotypic heterogeneity of *NRXN1*-related disorders.
